# RNAi for Plant Functional Genomics

**DOI:** 10.1002/cfg.396

**Published:** 2004-04

**Authors:** Louisa Matthew

**Affiliations:** CSIRO Plant Industry GPO Box 1600 ACT Canberra 2601 Australia

## Abstract

A major challenge in the post-genome era of plant biology is to determine the functions of all the genes in the plant genome. A straightforward approach to this problem is to reduce or knock out expression of a gene with the hope of seeing a phenotype that is suggestive of its function. Insertional mutagenesis is a useful tool for this type of study, but it is limited by gene redundancy, lethal knock-outs, nontagged mutants and the inability to target the inserted element to a specific gene. RNA interference (RNAi) of plant genes, using constructs encoding self-complementary ‘hairpin’ RNA, largely overcomes these problems. RNAi has been used very effectively in *Caenorhabditis elegans* functional genomics, and resources are currently being developed for the application of RNAi to high-throughput plant functional genomics.


**Comparative and Functional Genomics**
*Comp Funct Genom* 2004; **5**: 240–244.
Published online in Wiley InterScience (www.interscience.wiley.com). **DOI:** 10.1002/cfg.396

**Conference Review**

### RNAi for plant functional genomics

Louisa Matthew*
*CSIRO Plant Industry, GPO Box 1600, Canberra, ACT 2601, Australia*

**Correspondence to: Louisa Matthew, CSIRO Plant Industry, GPO Box 1600, Canberra, ACT 2601, Australia. E-mail: Louisa.Matthew@csiro.au*

Received: 6 February 2004
Accepted: 6 February 2004

### Abstract

**A major challenge in the post-genome era of plant biology is to determine the functions of all the genes in the plant genome. A straightforward approach to this problem is to reduce or knock out expression of a gene with the hope of seeing a phenotype that is suggestive of its function. Insertional mutagenesis is a useful tool for this type of study, but it is limited by gene redundancy, lethal knock-outs, non-tagged mutants and the inability to target the inserted element to a specific gene. RNA interference (RNAi) of plant genes, using constructs encoding self-complementary 'hairpin' RNA, largely overcomes these problems. RNAi has been used very effectively in *Caenorhabditis elegans* functional genomics, and resources are currently being developed for the application of RNAi to high-throughput plant functional genomics.**
**Copyright © 2004 John Wiley & Sons, Ltd.**

**Keywords:** RNA interference; post-transcriptional gene silencing; high-throughput; functional genomics

### Introduction

Plant genome and EST sequencing efforts have yielded an abundance of genetic information, including the complete genomic sequences of *Arabidopsis thaliana* and rice. Genome sequencing projects are also currently under way for maize, *Medicago truncatula* and *Lotus japonicus*, among others. In addition, a large number of EST sequencing projects are being pursued in a variety of plants. This wealth of information has created a need for efficient, high-throughput methods to utilize such data for functional genomic analyses. This need is well illustrated by the fact that less than 10% of the predicted 28 581 genes in the *Arabidopsis* genome [39] have been experimentally characterized, and 30% lack significant homology to characterized genes from any species [2].

In plants, RNA interference [RNAi, also known as post-transcriptional gene silencing (PTGS) or co-suppression] is thought to be a key defence against viruses (reviewed in [44]), as well as a way of regulating endogenous genes (reviewed in [22,24]). One key feature of RNAi is the production of double-stranded RNA (dsRNA) homologous to the gene being targeted for silencing [42]. This dsRNA is degraded into approximately 21-nucleotide RNAs, known as 'small interfering RNAs' (siRNAs), by the enzyme Dicer. These siRNAs then provide specificity to the endonuclease-containing, RNA-induced silencing complex (RISC), which targets homologous RNAs for degradation (recently reviewed in [17,32,40]). The effectiveness of RNAi for functional genomics has already been demonstrated in the nematode *Caenorhabditis elegans,* but has yet to be fully utilized in plants.

### Functional genomics in *C. elegans*

Functional genomics in *C. elegans* has progressed further than that of the model plant *Arabidopsis*. In *C. elegans*, several high-throughput reverse-genetic screens have been based on using RNAi to interrupt the expression of targeted genes [12,14,29], and

Copyright © 2004 John Wiley & Sons, Ltd.

recently the knock-down phenotypes of approximately 86% of the predicted *C. elegans* genes were analysed using this approach [23]. From these studies, it is clear that RNAi is a precise method for investigating gene functions in *C. elegans*. The primary advantage of RNAi in these studies is the simplicity of the techniques involved — feeding *C. elegans* with *E. coli* expressing dsRNA [41] or simply soaking worms in dsRNA [38] will knock down the homologous *C. elegans* gene. Results from large-scale RNAi investigations appear to integrate well with other large-scale approaches, specifically transcription profiling with microarrays and yeast two-hybrid studies of protein–protein interactions [15,25]. From these coordinated efforts, comprehensive pictures of the *C. elegans* genes involved in important biochemical and morphogenetic pathways are emerging.

### Mutagenesis-based functional genomics in plants

In contrast to the situation in *C. elegans*, high-throughput expression modulation techniques are not so easily applicable to plants. Current techniques of altering gene expression and function include chemical mutagenesis using ethylmethane sulphonate (EMS), insertional mutagenesis using transposons or T-DNAs and RNAi (reviewed in [4]). The more recently developed techniques utilizing RNAi will be discussed below. Chemical and insertional mutagenesis approaches have been applied extensively to the functional characterization of plant genes, and many successful examples of their uses have been published. However, these approaches have disadvantages stemming from the randomness of mutagenesis. Mutations must be sequenced or mapped to confirm their positions, and large collections of mutant lines are necessary to obtain good coverage of whole genomes. These limitations mean that the application of mutagenesis on a genomic scale is very labour-intensive.

Insertional mutagenesis, rather than chemical mutagenesis, is more commonly used in large-scale plant functional genomics, as mobile DNA tags of known sequence can be utilized to retrieve flanking gene sequences. Several databases of T-DNA and transposon insertion lines have been compiled, e.g. the Syngenta *Arabidopsis* Insertion Library (SAIL), which contains T-DNA flanking sequences from 52 964 lines [34], and the Salk Institute Genomic Analysis Laboratory (SIGnAL) collection of T-DNA insertions [33], which contains 88 122 T-DNA insertions, covering approximately 74% of the predicted *Arabidopsis* genes [1]. The large T-DNA insertion collections required to achieve moderate coverage of *Arabidopsis* genes reveal the primary limitation of random mutagenesis — that it cannot be targeted to specific plant DNA sequences. The possibility of multiple transposon or T-DNA insertions confounding the analysis of mutant lines is a further problem of insertional mutagenesis, e.g. lines in the SIGnAL collection have an average of about 1.5 T-DNA insertions/line [1] and in the SAIL collection an average of 1.5–2 T-DNA insertions/line [37]. Also, the fact that recessive mutations will only manifest their phenotypes in segregating M2 populations further complicates large-scale analysis of insertion lines.

The limitations arising from the randomness of mutagenesis are exemplified by the current coordinated effort to characterize all of the R2R3-*MYB* gene family members using insertional mutants [26,30,36]. There are 125 members of the R2R3 sub-family of *MYB* transcription factors in *Arabidopsis* [36]. By screening for T-DNA and transposon insertions in 73 of these genes, 47 insertions in 36 different target genes were identified [30]. This involved laborious screening — over 50 000 mutagenized lines were screened for insertions in subsets of the *MYB* genes. Therefore, while insertional mutagenesis has been shown to be very effective in terms of revealing mutant phenotypes, its applicability to high-throughput reverse-genetic analysis of plants is limited.

### RNAi for functional genomics in plants

In contrast to insertional mutagenesis, the interruption of gene expression by RNAi has only been utilized for plant functional genomics relatively recently. As discussed above, dsRNA triggers the degradation of homologous RNAs, such as mRNAs, thus preventing gene expression. dsRNAs are efficiently produced by intron-spliced hairpin transgenes (sense and antisense copies of the target gene separated by an intron), which have been

Copyright © 2004 John Wiley & Sons, Ltd.

found to be extremely effective in triggering RNAi [35]. dsRNAs can be delivered to plants in several ways (reviewed in [43]): microprojectile bombardment with dsRNA or intron-containing hairpin RNA (ihpRNA)-expressing vectors; infiltration of plant tissue with an *Agrobacterium* strain carrying a T-DNA expressing an ihpRNA transgene; virus-induced gene silencing (VIGS), in which the target sequence is integrated into viral sequences which are used to infect the plant, or are expressed from *Agrobacterium*-introduced transgenes, and by stable transformation with ihpRNA expressing transgenes. The various RNAi techniques each have advantages and disadvantages with respect to how persistent their effect is and the range of plants to which they can be applied, e.g. bombardment can be applied to any plant, but produces only transient effects. Alternatively, transformation with ihpRNA-expressing transgenes provides stable and heritable gene silencing, but requires efficient plant transformation techniques. ihpRNA transgenes have been shown to be very effective for a wide range of target genes in various plant species (reviewed in [43,45]), indicating that the RNAi mechanism is probably conserved in all plant species. This is supported by a recent report of RNAi in the non-vascular moss *Physcomitrella patens* [5].

RNAi has several advantages over insertional mutagenesis strategies for functional genomics, the primary advantage being an ability to specifically target the chosen gene. As RNAi is a homology-dependent process: careful selection of a unique region of the target sequence can ensure that a specific gene family member is silenced, or multiple members of a gene family can be silenced by targeting highly conserved sequence domains. In this way, redundancy is not limiting. RNAi can also be used to analyse the functions of essential genes. Variable levels of gene silencing can be achieved in different transgenic lines using the same ihpRNA construct [45], allowing selection of lines with a greater or lesser degree of silencing. Also, the expression of ihpRNAs from inducible promoters can control the extent and timing of gene silencing [7,16], such that essential genes are only silenced at chosen growth stages or in chosen plant organs. In these ways, RNAi provides the flexibility necessary for the characterization of genes of diverse functions.

### RNAi tools for functional genomics

Comprehensive resources for functional genomics in plants are being developed within the research community, the most notable examples being the sequencing of the *Arabidopsis* and rice genomes [2,13,46]. Utilization of the *Arabidopsis* genome is being made possible through efforts such as that of the complete *Arabidopsis* transcriptome microarray (CATMA) project [10], which aims to make a high-specificity gene sequence tag (GST) library covering all *Arabidopsis* genes. Compatible tools are also becoming available, such as Curtis and Grossniklaus' suite of binary vectors suitable for constitutive or inducible ectopic expression and expression of GUS or GFP fusion proteins [11], the pHELLSGATE high-throughput gene silencing vectors [19,20] and a high-throughput tobacco rattle virus-based VIGS vector [27]. An increasingly common feature, shared by all of these vectors, is the use of Invitrogen's Gateway recombination-based cloning technology [18] to replace previously time-limiting conventional cloning steps. The compatibility arising from common use of the Gateway system is currently being exploited by the *Arabidopsis* genome RNAi knock-out line analysis (AGRIKOLA) consortium to create a pHELLSGATE-based silencing vector for each GST in the CATMA collection, with the aim of producing ihpRNA constructs for all *Arabidopsis* genes [21]. These vectors will provide an invaluable resource for *Arabidopsis* functional genomics.

### Challenges for the use of RNAi for functional genomics in plants

There are limitations to the ways in which RNAi can currently be used for high-throughput functional genomics across different plant species. First, unlike insertional mutagenesis, knowledge of the target gene sequence is required. However, once sequence information is available, high-throughput approaches can be quickly applied, as is evident with the resources currently being developed for *Arabidopsis* functional genomics. With the increasing number of genome and EST sequencing projects, sequence knowledge is becoming much less of a limitation. Second, the methods of delivering dsRNA limit the species to which high-throughput approaches can be applied, e.g. transformation is necessary for stable expression of

Copyright © 2004 John Wiley & Sons, Ltd. 

ihpRNA transgenes, and *Arabidopsis* remains the only plant that can be transformed with the ease necessary for high-throughput approaches [9]. Similarly, VIGS, the main alternative to stable transformation with ihpRNA transgenes, still requires viral vectors compatible with the plant species of choice. To this end, improvements in plant transformation techniques and the development of further VIGS vectors is necessary.

The difficulty of detecting subtle or conditional mutant phenotypes has been reported for large-scale *C. elegans* reverse-genetic screens [12,23]. As could be expected in such studies, the efficiency of detection of mutant phenotypes depends upon how closely, and under what conditions, mutants are examined. Thus, a report of no phenotype is far from confirmation of a gene being redundant or non-functional. For plant screens, several approaches are being used to address this problem, including the development of standards of normal growth and development across a range of *Arabidopsis* growth stages [6] and the development of sets of growth conditions (with varied environmental stresses) to test for conditional mutants [3]. Inclusion of marker genes in ihpRNA constructs to indicate the strength of gene silencing presents a novel way of screening for subtle phenotypes using RNAi. Such an approach is based upon the discovery that inclusion of sequences from two genes within the one hairpin transgene results in their coordinate silencing [20]. By including a readily-visualized phenotypic marker, such a system could be used to identify strongly silenced lines which do not display obvious phenotypes. These can then be examined in more detail under varied environmental conditions.

### Conclusions

With the comprehensive resources currently being developed, RNAi-based functional genomics is quickly becoming a reality, particularly in *Arabidopsis*. The utility of RNAi strategies for functional genomics has been demonstrated by one of the first large-scale screens published, which utilized VIGS to investigate the function of almost 5000 random *Nicotiana benthamiana* cDNAs in *Pto*-mediated disease resistance [28]. However, more random and extensive screens are clearly necessary to investigate the functions of unclassified or uncharacterized genes. Challenging goals are being set for plant functional genomics by projects such as REGIA [31], which aims to characterize all *Arabidopsis* transcription factors, and the 2010 Project [8], which aims to functionally characterize all *Arabidopsis* genes by the year 2010. RNAi clearly provides a significant tool for the accomplishment of these goals, and will undoubtedly be used to address many other challenges in plant functional genomics.

### References

1. Alonso JM, Stepanova AN, Leisse TJ, *et al.* 2003. Genome-wide insertional mutagenesis of *Arabidopsis thaliana*. *Science* **301**: 653–657.
2. *Arabidopsis* Genome Initiative. 2000. Analysis of the genome sequence of the flowering plant *Arabidopsis thaliana*. *Nature* **408**: 796–815.
3. *Arabidopsis* Gantlet Project (accessed 23 January 2004): **http://thale.biol.wwu.edu/index/html**
4. Bevan M. 2002. Genomics and plant cells: application of genomics strategies to *Arabidopsis* cell biology. *Phil Trans R Soc Lond B Biol Sci* **357**: 731–736.
5. Bezanilla M, Pan A, Quatrano R. 2003. RNA interference in the moss *Physcomitrella patens*. *Plant Physiol* **133**: 470–474.
6. Boyes D, Zayed A, Ascenzi R, *et al.* 2001. Growth stage-based phenotypic analysis of *Arabidopsis*: a model for high-throughput functional genomics in plants. *Plant Cell* **13**: 1499–1510.
7. Chen S, Hofius D, Sonnewald U, Bornke F. 2003. Temporal and spatial control of gene silencing in transgenic plants by inducible expression of double-stranded RNA. *Plant J* **36**: 731–740.
8. Chory J, Ecker J, Briggs S, *et al.* 2000. Functional genomics and the virtual plant. A blueprint for understanding how plants are built and how to improve them. *Plant Physiol* **123**: 423–425.
9. Clough S, Bent A. 1998. Floral dip: a simplified method for *Agrobacterium*-mediated transformation of *Arabidopsis thaliana*. *Plant J* **16**: 735–743.
10. Crowe M, Serizet C, Thareau V, *et al.* 2003. CATMA: a complete *Arabidopsis* GST database. *Nucleic Acids Res* **31**: 156–158.
11. Curtis MD, Grossniklaus U. 2003. A gateway cloning vector set for high-throughput functional analysis of genes in planta. *Plant Physiol* **133**: 462–469.
12. Fraser AG, Kamath RS, Zipperlen P, *et al.* 2000. Functional genomic analysis of *C. elegans* chromosome I by systematic RNA interference. *Nature* **408**: 325–330.
13. Goff SA, Ricke D, Lan T-H, *et al.* 2002. A draft sequence of the rice genome (*Oryza sativa* L. ssp. *japonica*). *Science* **296**: 92–100.
14. Gonczy P, Echeverri C, Oegema K, *et al.* 2000. Functional genomic analysis of cell division in *C. elegans* using RNAi of genes on chromosome III. *Nature* **408**: 331–336.
15. Grant BD, Wilkinson HA. 2003. Functional genomic maps in *Caenorhabditis elegans*. *Curr Opin Cell Biol* **15**: 206–212.

Copyright © 2004 John Wiley & Sons, Ltd. 

16. Guo H-S, Fei J-F, Xie Q, Chua N-H. 2003. A chemical-regulated inducible RNAi system in plants. *Plant J* **34**: 383–392.
17. Hannon GJ. 2002. RNA interference. *Nature* **418**: 244–251.
18. Hartley JL, Temple GF, Brasch MA. 2000. DNA cloning using *in vitro* site-specific recombination. *Genome Res* **10**: 1788–1795.
19. Helliwell C, Waterhouse P. 2003. Constructs and methods for high-throughput gene silencing in plants. *Methods* **30**: 289–295.
20. Helliwell C, Wesley S, Wielpolska A, Waterhouse P. 2002. High-throughput vectors for efficient gene silencing in plants. *Funct Plant Biol* **29**: 1217–1225.
21. Hilson P, Small I, Kuiper M. 2003. European consortia building integrated resources for *Arabidopsis* functional genomics. *Curr Opin Plant Biol* **6**: 426–429.
22. Hunter C, Poethig R. 2003. Missing links: miRNAs and plant development. *Curr Opin Genet Dev* **13**: 372–378.
23. Kamath RS, Fraser AG, Dong Y, *et al.* 2003. Systematic functional analysis of the *Caenorhabditis elegans* genome using RNAi. *Nature* **421**: 231–237.
24. Kidner C, Martienssen R. 2003. Macro effects of microRNAs in plants. *Trends Genet* **19**: 13–16.
25. Kim SK. 2001. **Http://C. elegans**: mining the functional genomic landscape. *Nature Rev Genet* **2**: 681–689.
26. Kranz H, Denekamp M, Greco R, *et al.* 1998. Towards functional characterization of the members of the *R2R3-MYB* gene family from *Arabidopsis thaliana*. *Plant J* **16**: 263–276.
27. Liu Y, Schiff M, Dinesh-Kumar SP. 2002. Virus-induced gene silencing in tomato. *Plant J* **31**: 777–786.
28. Lu R, Malcuit I, Moffett P, *et al.* 2003. High throughput virus-induced gene silencing implicates heat shock protein 90 in plant disease resistance. *EMBO J* **22**: 5690–5699.
29. Maeda I, Kohara Y, Yamamoto M, Sugimoto A. 2001. Large-scale analysis of gene function in *Caenorhabditis elegans* by high-throughput RNAi. *Curr Biol* **11**: 171–176.
30. Meissner R, Jin H, Cominelli E, *et al.* 1999. Function search in a large transcription factor gene family in *Arabidopsis*: assessing the potential of reverse genetics to identify insertional mutations in R2R3 *MYB* genes. *Plant Cell* **11**: 1827–1840.
31. Paz-Ares J and consortium TR. 2002. REGIA, an EU project on functional genomics of transcription factors from *Arabidopsis thaliana*. *Comp Funct Genom* **3**: 102–108.
32. Pickford A and Cogoni C. 2003. RNA-mediated gene silencing. *Cell Mol Life Sci* **60**: 871–882.
33. Salk Institute Genomic Analysis Laboratory Homepage (accessed 23 January 2004): **http://signal.salk.edu**
34. Sessions A, Burke E, Presting G, *et al.* 2002. A high-throughput *Arabidopsis* reverse genetics system. *Plant Cell* **14**: 2985–2994.
35. Smith N, Singh S, Wang M-B, *et al.* 2000. Total silencing by intron-spliced hairpin RNAs. *Nature* **407**: 319–320.
36. Stracke R, Werber M, Weisshaar B. 2001. The *R2R3-MYB* gene family in *Arabidopsis thaliana*. *Curr Opin Plant Biol* **4**: 447–456.
37. Syngenta Arabidopsis Insertion Library (accessed 23 January 2004): **http://www.tmri.org/pages/collaborations/garlic_files/SAILabout.html**
38. Tabara H, Grishok A, Mello C. 1998. RNAi in *C. elegans*: soaking in the genome sequence. *Science* **282**: 430–431.
39. The Institute for Genomic Research (accessed 23 January 2004): **http://www.tigr.org**
40. Tijsterman M, Ketting RF, Plasterk RH. 2002. The genetics of RNA silencing. *Annu Rev Genet* **36**: 489–519.
41. Timmons L, Fire A. 1998. Specific interference by ingested dsRNA. *Nature* **395**: 854.
42. Waterhouse P, Graham M, Wang M-B. 1998. Virus resistance and gene silencing in plants can be induced by simultaneous expression of sense and antisense RNA. *Proc Natl Acad Sci USA* **95**: 13 959–13 964.
43. Waterhouse P, Helliwell C. 2003. Exploring plant genomes by RNA-induced gene silencing. *Nature Genet* **4**: 29–38.
44. Waterhouse P, Wang M-B. 2001. Gene silencing as an adaptive defence against viruses. *Nature* **411**: 834–842.
45. Wesley S, Helliwell C, Smith N, *et al.* 2001. Construct design for efficient, effective and high-throughput gene silencing in plants. *Plant J* **27**: 581–590.
46. Yu J, Hu S, Wang J, *et al.* 2002. A draft sequence of the rice genome (*Oryza sativa* L. ssp. *indica*). *Science* **296**: 79–92.

Copyright © 2004 John Wiley & Sons, Ltd. 
